# Role of IL-6 inhibitor in treatment of COVID-19-related cytokine release syndrome

**DOI:** 10.7150/ijms.53564

**Published:** 2021-01-21

**Authors:** Peng Du, Jie Geng, Feng Wang, Xiaobo Chen, Zhiwei Huang, Yuliang Wang

**Affiliations:** 1 Clinical Laboratory of Emergency Medicine, Tianjin Union Medical Centre, Tianjin, China.; 2 Graduate School of Tianjin Medical University, Tianjin, China.; 3 Department of Genetics, School of Basic Medical Sciences, Tianjin Medical University, Tianjin, China.; 4 Unicell Life Science Development Co., Ltd, Tianjin, China.; 5 Department of Clinical Laboratory Medicine, the Second Hospital of Tianjin Medical University, Tianjin Institute of Urology, Tianjin, China.

**Keywords:** COVID-19, cytokine release syndrome, interleukin-6, interleukin-6 inhibitor.

## Abstract

Cytokine release syndrome (CRS) may be the key factor in the pathology of severe coronavirus disease 2019 (COVID-19). As a major driver in triggering CRS in patients with COVID-19, interleukin-6 (IL-6) appears to be a promising target for therapeutics. The results of inhibiting both trans- and classical- signaling with marketed IL-6 inhibitors (tocilizumab, siltuximab and sarilumab) in severe COVID-19 patients are effective based on several small studies and case reports thus far. In this review, we described the evidence of the IL-6 response in patients with COVID-19, clarified the pathogenesis of the role of IL-6-mediated CRS in severe COVID-19, and highlighted the rationale for the use of anti-IL-6 agents and key information regarding the potential features of these IL-6 inhibitors in COVID-19 patients.

## Introduction

On 11 March 2020, the World Health Organization (WHO) announced that novel coronavirus disease 2019 (COVID-19) was an outbreak that was first reported on 8 December 2019 in Hubei Province in China. This pandemic has accounted for ~85 million confirmed cases and ~ 1, 861,005 deaths (as of 7 Jan 2021) in more than 222 countries without even reaching the peak 1. North America is thus far the most heavily affected area, followed by Europe, Asia and South America. Although showing a lower case-fatality rate, the death toll from COVID-19 is higher than those from severe acute respiratory syndrome (SARS) and Middle East respiratory syndrome (MERS) combined 2, 3. The disease-related fatality associated with severe acute respiratory syndrome coronavirus 2 (SARS-CoV-2) infections in humans can be attributed in part to life-threatening hyperinflammation sustained by a cytokine storm that eventually leads to multiple organ failure 4. This hyperinflammatory response is associated with elevated levels of inflammatory cytokines, including interleukin-6 (IL-6) 5. Recent data indicate that IL-6 plays an important role in the COVID-19-related cytokine release storm (CRS) 6. Thus, according to international guidelines, IL-6 inhibitors are recommended as one of the options available to severe or critically ill patients 7. There has been increased interest in employing the inhibitory agents tocilizumab, siltuximab and sarilumab in COVID-19 patients, with a series of clinical trials being registered and launched in different countries. In addition, the use of anti-IL-6 drugs is associated with an increased risk of some adverse reactions 8. The use of metalloproteinase 17 (ADAM17) or soluble glycoprotein 130 fused chimera (sgp130Fc) to specifically inhibit pathological IL-6 trans-signaling in patients with severe COVID-19 promises to be a more effective and safer strategy to ameliorate disease states. Therefore, this review aims to summarize the available and latest research progress on the association between SARS-CoV-2-induced CRS and IL-6 and the development of IL-6 inhibitors.

## IL-6: A Double-Edged Sword in Inflammation

IL-6 can be produced by almost all stromal and immune system cells, including B lymphocytes, T lymphocytes, macrophages, monocytes, dendritic cells, mast cells, and other nonlymphocytic cells, such as fibroblasts, endothelial cells, keratinocytes, glomerular mesangial cells and tumor cells. As a multifunctional member of the cytokine network, IL-6 acts as a mediator between pro- and anti-inflammatory reactivity by initiating trans- and classical signaling, respectively 9. In addition, IL-6 is responsible for bridging the gap between the innate and adaptive arms of the immune response 10, 11.

### Signal Transduction Pathway of IL-6

The signaling mechanisms employed by IL-6 are highly complex; therefore, it is often challenging to understand how IL-6 receptor signaling can create a diverse range of biological responses. There are three main modes of IL-6 signal transduction 9, 12: classical signal transduction, trans-signal transduction, and a newly reported mechanism called trans-presentation. In the classical signal transduction pathway, IL-6 binds to the membrane-bound IL-6 receptor (mIL-6R) to build a complex 13. Then, the monomeric complex of IL-6 and mIL-6R associates with ubiquitously expressed transmembrane glycoprotein 130 (gp130), which results in gp130 dimerization. IL-6R exists not only in the transmembrane form but also in a soluble form (sIL-6R). IL-6 binds to these two forms and then interacts with gp130 to initiate intracellular signal transduction and gene expression 14. sIL-6R can also bind to and activate gp130 on cells that do not carry mIL-6R, thereby enhancing the spectrum of target cells for IL-6, which explains the pleiotropic functions of IL-6. In a healthy state, classical IL-6 receptor signaling is protective and controls various metabolic processes and tissue renewal or regeneration 9. IL-6 trans-signaling is associated more widely with the regulation of inflammatory processes relevant to disease. A third signal mode, termed IL-6 trans-presentation, is a juxtracrine mechanism of IL-6 signaling that promotes the engagement of dendritic cells with T cells 15, which may lead to priming of Th17 cells in combination with transforming growth factor beta 2 (TGF-β2), induction of the generation of pathogenic Th17 cells, (chronic) inflammation, and suppression of Treg cells by IL-27. The next process is to activate the Janus kinase/signal transducer and activator of transcription (JAK-STAT) pathway 16; Additionally, the RAS-RAF 17, SRC-YAP-NOTCH 18 and AKT-PI3K 19 pathways are activated. These processes enable complex biological functions such as proliferation, differentiation, and immune regulation to be realized 20.

### Pro- and Anti-inflammatory Effects of IL-6

IL-6 is the main force that triggers the proinflammatory response during infection. The trans-signaling of IL-6 is significantly positively correlated with the production of proinflammatory cytokines and chemokines 10. In addition, IL-6 trans-signaling can also stimulate the differentiation of monocytes into macrophages, attract other immune cells, and restrain the function of Treg cells 21. When infections or tissue injuries occur, IL-6 is promptly produced by monocytes and macrophages and contributes to the removal of infectious agents and restoration of damaged tissues through activation of immune, hematological, and acute-phase responses. Once stress is removed from the host, IL-6 synthesis finishes, but uncontrolled excessive or persistent IL-6 production plays a pathological role in the development of acute severe inflammatory diseases or chronic diseases and cancers 22.

Though often described as a proinflammatory molecule, IL-6 has demonstrated protective properties from a substantial body of evidence. Recent research has indicated a potentially inflammation-suppressing effect of IL-6 in the lungs. The study showed that lung inflammation and injury were more likely to occur in IL-6 knockout (KO) mice exposed to lipopolysaccharide (LPS) than in wild-type (WT) mice 23. These IL-6 KO mice given exogenous recombinant IL-6 had less severe lung injury and less pulmonary edema than KO mice not given recombinant IL-6. IL-6 has demonstrated a protective role in the host response to infection. A recent study published in *Nature* showed that IL-6 KO mice were impaired in response to vaccinia virus, with a 10~1000-fold increase in viral titers in IL-6-deficient animals 24. These IL-6 KO mice also exhibited increased susceptibility to Listeria monocytogenes infection, possibly because of the reduced bactericidal activity of macrophages. In IL-6 KO mice exposed to inhaled Streptococcus pneumoniae, an increased number of pulmonary S. pneumoniae bacterial colonies and reduced survival time relative to WT mice were observed 25. Moreover, IL-6 enhanced epithelial cell survival and promoted the migration and survival of macrophages. Interestingly, a protective role for IL-6 has been shown in coinfection with influenza virus and S. pneumoniae bacteria. Likewise, IL-6 was elevated during coinfection and was the most prominent of all cytokines measured. Together, these data emphasize a vital role for IL-6 in the host immune response to infection. This suggests that blocking IL-6 activity might not promote but diminish host defense against viral or bacterial lung infections.

### ADAM17: the Manufacturer of sIL-6R

The ADAM family member ADAM17 is a type I transmembrane protease that drives the limited proteolysis of over 80 cell membrane-bound cytokines, chemokines, growth factors, adhesion molecules, and their receptors 26. ADAM-17 is considered to be the key molecule that may explain uncontrolled IL-6 trans-signaling and increased proinflammatory responses during infection. This is because ADAM-17 is the major protein that leads to mIL-6R shedding and, thus, the production of sIL-6R 11. Apoptosis has been proven to be a natural stimulus in ADAM-17-mediated IL-6R shedding from the surface of neutrophils and thereby contributes to proinflammatory trans-signaling responses 27, 28. Uncontrolled IL-6 trans-signaling could also be explained by this mechanism, from which a picture of how infection worsens can be envisioned. Therefore, ADAM-17 should be thought of as a potential target molecule for novel antiviral drug discovery that can regulate host reactivity to infection and, in turn, limit or prevent fatal outcomes.

## Role for IL-6-mediated CRS of Severe COVID-19

Huang et al. 6 reported the clinical features and cytokine profile of critically ill patients with COVID-19 in Wuhan, China, and suggested that a cytokine storm, also known as cytokine release syndrome (CRS), could be associated with disease severity. After virus infection, dendritic cells, macrophages, and neutrophils, as the first line of defense, start the immune reaction and affect its type and intensity. Autopsies on patients who died of COVID-19 revealed a high infiltration of macrophages within the area of bronchopneumonia 29. These macrophages significantly produce IL-6, suggesting that they may be the cause of excessive inflammation in COVID-19 disease 30. Similarly, in SARS disease, which represents the closest disease to COVID-19 in humans, high production of IL-6 was also previously described. SARS produces even more intense IL-6 than common viral respiratory diseases (e.g., influenza and parainfluenza) 31. Recent studies have implied the possibility that inflammatory cytokine storms and inflammatory events may be responsible for severe COVID-19 pathology 32. Thus, IL-6 should not be ignored in the treatment of severe COVID-19.

According to a recent meta-analysis, significantly higher levels of IL-6 in serum are demonstrated to be predictors of the disease severity and prognosis of patients with COVID-19 33. Likewise, another meta-analysis indicated that elevated IL-6 levels occur more often in severe and critically ill COVID-19 patients than in mildly ill COVID-19 patients, and they occur more often in patients who die from the disease than in those who survive 34. This might help clinicians identify critical patients in a timelier and more effective manner.

However, before regarding IL-6-mediated CRS as the pathological driver of severe COVID-19, caution should be warranted. It is noteworthy that COVID-19 patients lack most of the hallmarks of CRS, including hypotension, capillary leak syndrome, and neurotoxicity 35. In addition, the clinical course of CRS is much more acute than that of COVID-19 36. Evidence shows that, compared with 1,000-10,000 pg/ml in CRS, serum IL-6 levels are far lower in COVID-19, with peak levels typically less than 100 pg/ml in COVID-19 33, 36.

## IL-6 Inhibition as a Strategy for the Treatment of COVID-19-related CRS

In view of the key role of IL-6 in COVID-19-related CRS, neutralizing antibodies used to treat a number of autoimmune diseases by targeting the exacerbated inflammatory immune response of the host may provide a life-saving approach by preventing cytokine release syndrome in severe cases. Tocilizumab, a specific monoclonal antibody that blocks IL-6, has been recommended for use in severely or critically ill patients with extensive lesions in bilateral lungs and confirmed elevated levels of IL-6 in the Diagnosis and Treatment Protocol for Novel Coronavirus Pneumonia (Trial Version 8) issued by the National Health Commission of China on August 19th, 2020 37. Tocilizumab is also mentioned in the NIH treatment guidelines and the WHO interim guidance, both of which cite insufficient clinical data to recommend either for or against the use of these drugs, the same as for other drugs 7, 38. At present, more IL-6 inhibitors, such as sarilumab and siltuximab, are utilized in the treatment of COVID-19 based on the biology of IL-6.

### Tocilizumab (Actemra)

Tocilizumab is a humanized anti-IL-6R monoclonal antibody. Several cohort studies demonstrated the efficacy of tocilizumab in treating severe cases of COVID-19. A Chinese retrospective observational study reported a case series of patients with COVID-19 treated with tocilizumab 39; 46.7% of these patients were critically ill. The study authors summarize that tocilizumab might be an applicable treatment with high IL-6. Xu et al 40 reported 21 patients diagnosed with severe or critical COVID-19. Twenty of those 21 patients recovered and were discharged within 2 weeks after tocilizumab therapy, with no adverse drug events. The authors reported that the body temperatures of all patients returned to normal values within the first day after receiving tocilizumab and remained stable thereafter. By the fifth day of treatment, 75% of patients had a reduced need for oxygen supplementation, and one patient needed no oxygen therapy. In a prospective open, single-arm study, 63 patients with severe cases of COVID-19 were administered tocilizumab either once or twice with doses taken 24 h apart 41. In one day, marked reductions in fever were observed in all patients except one, and the levels of C-reactive protein (CRP), ferritin, and D-dimer and the lymphocyte count were improved in most patients. In France, an open-label Phase II RCT (CORIMUNO-TOCI, NCT04331808) is being conducted by the public assistance hospitals of Paris 42. The interim results of this study were released recently through press release. Sixty-five of the 129 patients were randomized to receive tocilizumab 8 mg/kg (1-2 injections) along with standard of care, and 64 received standard of care alone. A significantly lower proportion of the patients in the tocilizumab arm attained the primary outcome of the need for ventilation or death at day 14. These results showed positive effects of tocilizumab and hinted that IL-6 might be an ideal therapeutic target for COVID-19 patients with CRS.

Another prospective study was conducted in Italy on 100 COVID-19 patients with acute respiratory failure 43. They received two doses of tocilizumab every 12 h. Within ten days, the respiratory conditions of 77 patients were significantly improved. However, the respiratory function of 23 patients deteriorated, 20 of them died, and three patients experienced serious adverse events. Similarly, a retrospective controlled study supports that tocilizumab is not effective in modifying the 30-day mortality of patients with severe and critical COVID-19 with hyperinflammation 44. It was recently shown that treatment with tocilizumab resulted in poor outcomes in COVID-19 patients and did not prevent them from developing secondary hemophagocytic lymphohistiocytosis 45. Genentech, the innovator company of tocilizumab, has also initiated a randomized, double-blind, placebo-controlled Phase III clinical trial (COVACTA, NCT04320615) to evaluate the safety and efficacy of tocilizumab plus standard of care in hospitalized adult patients with severe COVID-19 pneumonia compared with placebo plus standard of care 46. The trial aims to recruit approximately 330 patients globally. Unfortunately, the COVACTA trial did not meet its primary endpoint of improved clinical status in hospitalized adult patients with severe COVID-19-associated pneumonia. In addition, the key secondary endpoints, which included the difference in patient mortality at week four, were not met; however, there was a positive trend in time to hospital discharge in patients treated with tocilizumab. The COVACTA study did not identify any new safety signals for tocilizumab. More prospective controlled randomized clinical trials are expected to evaluate whether tocilizumab is still considered an anti-inflammatory treatment for COVID-19, and further studies should focus on the optimal timing of treatment. The stage of disease may also affect the efficacy of IL-6 blockade therapy.

### Sarilumab (Kevzara)

Sarilumab is a fully human IgG1 monoclonal antibody that targets both IL-6R and mIL-6R, thus inhibiting both classical and trans-IL-6-mediated inflammatory pathways 47. A recent study showed that sarilumab has exhibited promising potential in improving the symptoms of COVID-19 patients. The addition of sarilumab to the therapy regimen of eight hospitalized COVID-19-positive patients showed significant improvement in respiratory functions by reducing oxygen demand by 30%. Moreover, early and aggressive intervention in these patients with IL-6 blockers resulted in their discharge after 14 days of hospitalization, and seven out of them tested back negative for COVID-19 48. In the USA, the 'Evaluation of the Efficacy and Safety of Sarilumab in Hospitalized Patients With COVID-19' (NCT04315298) was launched, which is a phase II/III, randomized, double-blind, placebo-controlled clinical trial, for 400 patients recruited in March 2020 by Sanofi and Regeneron 49. On July 2nd, 2020, Regeneron Pharmaceuticals, Inc. (NASDAQ: REGN) and Sanofi announced that the U.S. phase 3 trial of Kevzara® (sarilumab) 400 mg in COVID-19 patients requiring mechanical ventilation did not meet its primary and key secondary endpoints. Minor positive trends were observed in the primary prespecified analysis group that did not reach statistical significance, and these trends were countered by negative trends in a subgroup of critical patients who were not mechanically ventilated at baseline. In the primary analysis group, adverse events were experienced by 80% of Kevzara patients and 77% of placebo patients. Serious adverse events that occurred in at least 3% of patients and more frequently among Kevzara patients were multiorgan dysfunction syndrome (6% Kevzara, 5% placebo) and hypotension (4% Kevzara, 3% placebo). Based on the results, the U.S.-based trial was stopped, including in a second cohort of patients who received a higher dose of Kevzara (800 mg). More studies have since been registered or initiated for assessing sarilumab in the treatment of COVID-19.

### Siltuximab

Siltuximab is a chimeric human-mouse monoclonal antibody against IL-6 for the treatment of patients with multicentric Castleman disease (MCD) approved by the Food and Drug Administration (FDA) 50. It binds directly to soluble IL-6 and prevents it from acting on the IL-6 receptor to activate the IL-6 signaling pathways. Positive preliminary data from 21 COVID-19 patients admitted with respiratory complications have been reported by one center in Italy (NCT04322188), showing that after intravenous introduction of siltuximab, CRP levels were reduced markedly in most patients. Thirty-three percent of patients showed an improvement in their condition and no longer required ventilatory assistance, 43% were in a stable condition, and 24% experienced deterioration 51.

What is potentially important is that the clinical results of therapeutic inhibition of IL-6 with tocilizumab or sarilumab are contradictory at this point, at least inferring that in many cases, IL-6-led CRS may not be the main cause of COVID-19-related morbidity and mortality 51. This may be partly due to the continued production of IL-6 overcoming the direct targeting effect 52. The failure of the phase III clinical trials of two IL-6 receptor inhibitors, tocilizumab and sarilumab, in the treatment of severe new coronary pneumonia shows that blocking this pathway may only have some efficacy in the most severe patients. These results suggest that in the treatment of critically ill patients, inhibition of only the IL-6 pathway in CRS may not be enough. It may also be necessary to pay attention to other pathways that cause CRS or to combine treatment with antiviral drugs. Combination therapy from multiple aspects may be a promising strategy for the effective treatment of COVID-19. Moreover, in the case of viral infections such as COVID-19, macrophage activation occurs as a primary response to viral infection 53. Weakening the innate immune response may reduce off-target toxicity, and in the absence of other virus control (through drug therapy or immune-mediated control alternatives), it may cause more serious viral infections. In addition, IL-6 has significant anti-inflammatory properties. This allows people to question the principle of using IL-6 antagonists as a therapy to reduce inflammation. Studies have also shown that IL-6 performs an important function in the early host immune response to infection. Therefore, IL-6 blockade may not be an effective strategy to treat infection. The elevated level of circulating IL-6 in COVID-19 is usually not higher than the level of patients with autoimmune diseases, which raises questions about whether IL-6 is the causative cytokine in COVID-19 pulmonary or systemic injury. Finally, the clinical use of IL-6 inhibitors in autoimmune conditions increases the risk of serious infections (such as tuberculosis). Other common adverse reactions of anti-IL-6 agents include increased transaminase blood levels and cytopenia 54-56. These issues should arouse attention; that is, IL-6 inhibitors may be harmful in addition to being unlikely to benefit COVID-19 patients.

## The Prospect of Targeting Only sIL-6R-dependent Trans-signaling

It is noteworthy that trans-signaling via sIL6-R is believed to act in a rather proinflammatory way via recruitment of mononuclear cells, inhibition of T-cell apoptosis, and inhibition of regulatory T-cell differentiation 21. If we can block the proinflammatory effect of the trans-signaling pathway and retain the protective effect of classic IL-6 signal transduction, it may be possible to impede the otherwise uncontrolled proinflammatory response of severe SARS-CoV-2 infection. Sgp130Fc and an inhibitor of ADAM-17 may become a new “game changer” to block only the trans-signaling pathway as substitutes for tocilizumab.

Sgp130Fc, a fusion protein of the gp130 receptor with the Fc portion of a human immunoglobulin antibody, selectively inhibits the trans-signaling pathway 57. Therefore, sgp130Fc is expected to be a potential inhibitor of IL-6 in COVID-19 patients because it can retain the regenerative and anti-inflammatory properties of the IL-6 classic pathway and only block the proinflammatory effects mediated by the trans-signaling pathway 10. Using sgp130Fc in a murine polymicrobial sepsis model improved survival up to 100%. Evidence shows that using sgp130Fc to block IL-6 trans-signaling is as effective as using a specific monoclonal antibody to completely block IL-6 classic and trans-signaling 58. Thus, inhibition of only the proinflammatory pathway of IL-6 seems to be a more reasonable choice than inhibition of both pathways.

As we mentioned above, ADAM-17 is mostly responsible for mIL-6R shedding and producing sIL-6R and controls pro- and anti-inflammatory responses to viral antigenic stimuli. Several studies have suggested that the pathogenesis mediated by ADAM-17-induced IL-6 trans-signaling is common to inflammatory bowel disease and many respiratory diseases 59, 60. What may be inferred from this is that in severe SARS-CoV-2 infection, ADAM-17-induced IL-6 trans-signaling eventually interferes with the balance between pro- and anti-inflammatory responses and initiates IL-6 trans-signaling-mediated continuous pro-inflammatory responses. Highly selective and potent ADAM17 inhibitors such as A17pro are expected to be more effective in suppressing disease states related to IL-6 trans-signaling, with fewer adverse effects. Hence, developing a selective inhibitor of ADAM-17 for COVID-19 patients with CRS may be a promising strategy to alleviate inflammatory responses.

## Conclusion

COVID-19 has affected millions of people around the world. Thus, the need to find an effective therapeutic approach for serious viral infections or to reevaluate existing methods is more urgent than ever. Precision medicine is generally considered to have fewer side effects and more prominent effects, but only if we know what components are responsible. IL-6 may be the cause of excessive inflammation in SARS-CoV-2-induced CRS. Inhibiting both trans- and classical signaling with marketed IL-6 inhibitors (tocilizumab, siltuximab and sarilumab) in severe COVID-19 patients has been effective in several small studies and case reports thus far. In addition, we propose that a selective inhibitor of ADAM-17 or sgp130Fc may be a possible antiviral target. It may be necessary to conduct more experimental clinical studies to monitor the safety and efficacy of IL-6 inhibitors because it is urgent to give attention to those effectors that can prove to be "life-saving" treatment options.
